# Modelling net energy of commercial cat diets

**DOI:** 10.1371/journal.pone.0218173

**Published:** 2019-06-11

**Authors:** Natalie J. Asaro, David J. Seymour, Wilfredo D. Mansilla, John P. Cant, Ruurd T. Zijlstra, Kimberley D. Berendt, Jason Brewer, Anna K. Shoveller

**Affiliations:** 1 Centre for Nutrition Modelling, Department of Animal Biosciences, University of Guelph, Guelph, Ontario, Canada; 2 Centre for Genetic Improvement of Livestock, Department of Animal Biosciences, University of Guelph, Guelph, Ontario, Canada; 3 Department of Agricultural, Food and Nutritional Science, University of Alberta, Edmonton, Alberta, Canada; 4 Royal Canin, Mars Pet Care, Lewisburg, Ohio, United States of America; University of Illinois, UNITED STATES

## Abstract

Net energy accounts for the proportion of energy expenditure attributed to the digestion, metabolism, and absorption of ingested food. Currently, there are no models available to predict net energy density of food for domestic cats. Therefore, the objectives of this study were to measure the heat increment of feeding in cats, and to model the net energy of commercial diets. Metabolizable energy and calorimetry data from two previous studies was reanalyzed to create net energy models in the present study. Energy expenditure was calculated using measurements of CO_2_ production and O_2_ consumption. Net energy was determined as the metabolizable energy of the diets minus the heat increment of feeding. The heat increment of feeding was determined as the area under the energy expenditure curve above the resting fed metabolic rate. Eight net energy models were developed using metabolizable energy, 1 of 4 dietary parameters (crude protein, fat, fiber, and starch), and heat increment of feeding values from 0–2 h or 0–21 h. Two hours postprandial, and over the full calorimetry period, the heat increment of feeding amounted for 1.74, and 20.9% of the metabolizable energy, respectively. Of the models tested, the models using crude protein in combination with metabolizable energy as dietary parameters best fit the observed data, thus providing a more accurate estimate of dietary energy availability for cats.

## Introduction

Net energy (**NE**) models have been developed for use with multiple agricultural species such as swine and cattle [1, 2], but to date, they do not exist for the domestic cat. North American pet food industry standards currently use the modified Atwater equation to estimate the metabolizable energy (**ME**) of pet foods [3, 4]. This equation assigns coefficients to three macronutrients–protein, fat, and carbohydrate (calculated as nitrogen-free extract)–to predict the ME content of a diet [3]. However, these equations result in inaccurate predictions of dietary energy content [3]. Developing models to accurately predict the available energy density of food intended for cats is critical to provide consumers with optimal feeding recommendations [4].

Net energy can be described as the ME minus the heat increment of feeding (**HIF**) [5]. The HIF, also commonly referred to as dietary-induced thermogenesis, is variable and can be altered by dietary macronutrient content, diet processing conditions, and environmental temperature [6, 7]. Therefore, the HIF is important to measure for an accurate representation of dietary energy directly available to the animal [6]. The HIF can be measured through indirect calorimetry, which uses measures of oxygen and carbon dioxide exchange to calculate energy expenditure of animals [8]. Compared to ME, which accounts for fecal and urinary energy losses, NE is more complex to quantify. To overcome this issue, mathematical models have been developed that predict the NE content of feeds based on macronutrient composition [1, 2]. Unlike ME models, NE gives a precise estimate of the energy directly available for use by an animal [5]. Noblet et al. [1] proposed a model to estimate the NE of pig feed, using macronutrient composition and ME content of the complete diet as parameters. This model provides a superior prediction of the true energy content of a feed and allows for increased accuracy of formulation and subsequent feeding recommendations.

The objectives of this study were to calculate NE content of three diets differing in macronutrient profile, ingredient composition, and perceived glycemic responses (**PGR**), and to propose new models that can accurately predict NE of domestic cat diets. We hypothesized that 1) parameters used for NE models in other monogastric species can be used to predict NE of domestic cat diets; and 2) due to differences in macronutrient profile, ingredient composition and PGR, HIF would be different between diets.

## Materials and methods

Data from two previous studies were reanalyzed to create net energy models in the present study. Metabolizable energy data, analyzed by measuring fecal and urinary energy losses, was obtained from Asaro et al. [4], and calorimetry data was obtained from Asaro et al. [9], which were both conducted under Animal Utilization Protocol 013–9127 (dated 17 March 2013). The cats used for these different collection methods were purpose bred and trained for these specific methodologies and as such, we were unable to keep the same cohort of cats for both previously conducted studies. Therefore, to limit opportunity for differences in digestibility and energy expenditure measurements, both cohorts of cats were of similar age and BCS and were fed the same batches of the three commercial diets. All procedures were reviewed and approved by Proctor and Gamble Pet Care's Institutional Animal Care and Use Committee and were in accordance with the United States Department of Agriculture and the Association for Assessment and Accreditation of Laboratory Animal Care guidelines.

For further information regarding study design, such as animal housing and calorimetry, readers are referred to Asaro et al. [4, 9].

### Net energy modelling and statistical analyses

Calorimetry data from Asaro et al. [9] which included 57 observations (n = 19; 3 diets) was reanalyzed in the present study for the purpose of creating models to predict dietary net energy. Energy expenditure (**EE**) was calculated as *EE (kcal/d) = [3*.*94 x O*_*2*_ *exchange (L/h) + 1*.*11 CO*_*2*_ *exchange (L/h)] x 24 h* [10] and was segmented by time point, similar to Gooding et al. [11]. Fasted measurements were taken 60 and 30 min prior to feeding. Postprandial EE, fed EE, return to fasted EE, and late fasted EE were measured between 0–5.5 h, 5.5–10.5 h, 10.5–15.5 h, and 15.5–21 h post feeding, respectively. Since a distinct increase in EE was observed between 0 and 2 h, the average EE during the first 2 h post feeding was also analyzed. The resting fed metabolic rate (**RFMR**) was defined as the lowest observed value of energy expended by each animal [3].

In period 2, data was not recorded past the 10.5 h calibration for 4 cats due to software malfunction. Two of these cats were fed the LowPGR diet, one was fed the MediumPGR diet, and one was fed the HighPGR diet. The data that was collected in the 12 h previous to calibration was included in our analyses. In period 3, the calorimetry software did not record the first 12 h of data for one cat fed the HighPGR diet. Data from this cat was not included in our HIF calculations.

The NE of the diets was determined as the ME per 100 g dry matter, minus HIF associated with consuming the same amount of food [12]. Heat increment of feeding was calculated for the first 2 h post feeding and the entire calorimetry period (0–21 h), as the difference in area under the curve of postprandial EE minus the RFMR. Area under the curve was calculated using the linear trapezoidal rule [13].

Our proposed NE models were developed using both the HIF from 0–2 h postprandial, and the HIF for the complete calorimetry period (0–21 h). The pool of candidate predictor variables was selected to match the variables included in net energy prediction equations for swine, as suggested by Noblet et al [1]. Parameter estimates and fit statistics for each model were determined using 10-fold cross validation (seed = 495857) [14] and the PROC REG procedure in SAS (version 9.4; SAS Institute Inc., Cary, NC). Multicollinearity of predictors was declared when variation inflation factor > 10 [15]. The developed models were compared using root mean-square percentage error (**RMSPE)**, and MSPE values were decomposed into error in central tendency, error due to regression, and error due to random disturbance [16].

Statistical analyses were conducted using SAS version 9.4 (SAS Institute Inc., Cary, NC). Statistical power was calculated for EE and was determined to be 97.4% for the sample of 19 cats [10]. Correlations between calculated NE and analyzed ME were calculated using the CORR procedure. HIF data were analyzed using the MIXED procedure with individual cat as the experimental unit, diet as a fixed effect, and cat and period as random effects. Repeated measures analyses were performed for EE over time using a compound symmetry covariance structure, which was selected as it was associated with the lowest Akaike information criterion. The PDIFF option was specified to estimate differences in fixed effects. Energy expenditure was pooled from all diets, and differences in EE across time were compared against fasted EE (time = -30 min), using the MIXED procedure with a Dunnett test. Statistical significance was declared based on a Type I error rate of 0.05. Data were reported as least-squares means ± SEM.

## Results

### Diet composition, bodyweight & feed intake

The composition of the three test diets differed in macronutrient content (Table 1; [4]). Over the duration of the study, body weight did not differ among dietary treatments (*P* = 0.987). On an as-fed basis, daily food intake was higher for the HighPGR treatment (45.6 ± 2.7 g/d) than the MediumPGR (37.9 ± 2.7 g/d) and LowPGR treatments (40.0 ± 2.7 g/d) (Table 2; [9]). However, ME intake did not differ between diets, as intended (Table 2; [9]). Differences in energy intake would have required inclusion of energy intake on the HIF. Indeed, the effect of volume of food should be pursued in the future.

**Table 1:** Proximate analysis of commercial diets differing in PGR^1^.

Component	HighPGR^2^	MediumPGR^3^	LowPGR^4^
Moisture, %	7.16	6.76	5.31
Ash, %	6.36	6.31	6.38
Crude protein^5^, %	38.02	35.86	42.06
Crude fat, %	10.83	20.02	20.42
Nitrogen-free extract, %	34.1	29.5	23.6
Starch, %	36.75	30.72	23.56
Crude fiber, %	1.17	1.78	2.58

^1^Adapted from Asaro et al. [4] (S1 Table).

^2^Purina ONE Chicken and Rice (Nestlé, St. Louis, MO) containing as main ingredients: chicken, brewer’s rice, corn gluten meal, poultry by-product meal, wheat flour, animal fat preserved with mixed-tocopherols, whole grain corn, soy protein isolate, fish meal, animal liver flavor, KCl, H3PO4, CaCO3, caramel color, choline chloride, and salt.

^3^Iams Kitten Proactive Health (Procter & Gamble, Cincinatti, OH) containing as main ingredients: chicken, chicken by-product meal, corn meal, chicken fat preserved with mixed tocopherols, dried beet pulp, ground whole grain sorghum, dried egg product, natural flavor, fish oil preserved with mixed tocopherols, KCl, fructooligosaccharides, choline chloride, CaCO3, brewer’s dried yeast, DL-Met, and salt.

^4^Innova (Procter & Gamble, Cincinatti, OH) containing as main ingredients: turkey, chicken, chicken meal, whole grain barley and whole grain brown rice, chicken fat preserved with mixed tocopherols, peas, natural flavors, apples, herring, flaxseed, eggs, blueberries, pumpkin, tomatoes, sunflower oil, KCl, DL-Met, carrots, pears, cranberries, menhaden oil, cottage cheese, taurine, green beans, alfalfa sprouts, parsnips, and salt.

^5^Percentage of N × 6.25

**Table 2:** Bodyweight, food and energy intake for cats (n = 19) consuming three commercial diets differing in PGR^1^.

Variable	HighPGR	MedPGR	LowPGR	SEM
Bodyweight (kg)	4.99	4.95	4.94	0.33
Food intake (g/day)	45.6^a^	37.9^b^	40.0^b^	2.68
Calculated ME Intake (kcal/day)	155.8	154.5	154.3	10.08
Calculated ME Intake (kJ/day)	652.2	646.4	645.4	40.17

^1^Adapted from Asaro et al. [9] (S2 Table).

^a-b^Within a row, means without a common superscript differ (*P* < 0.05).

### Indirect calorimetry

**Fig 1:**
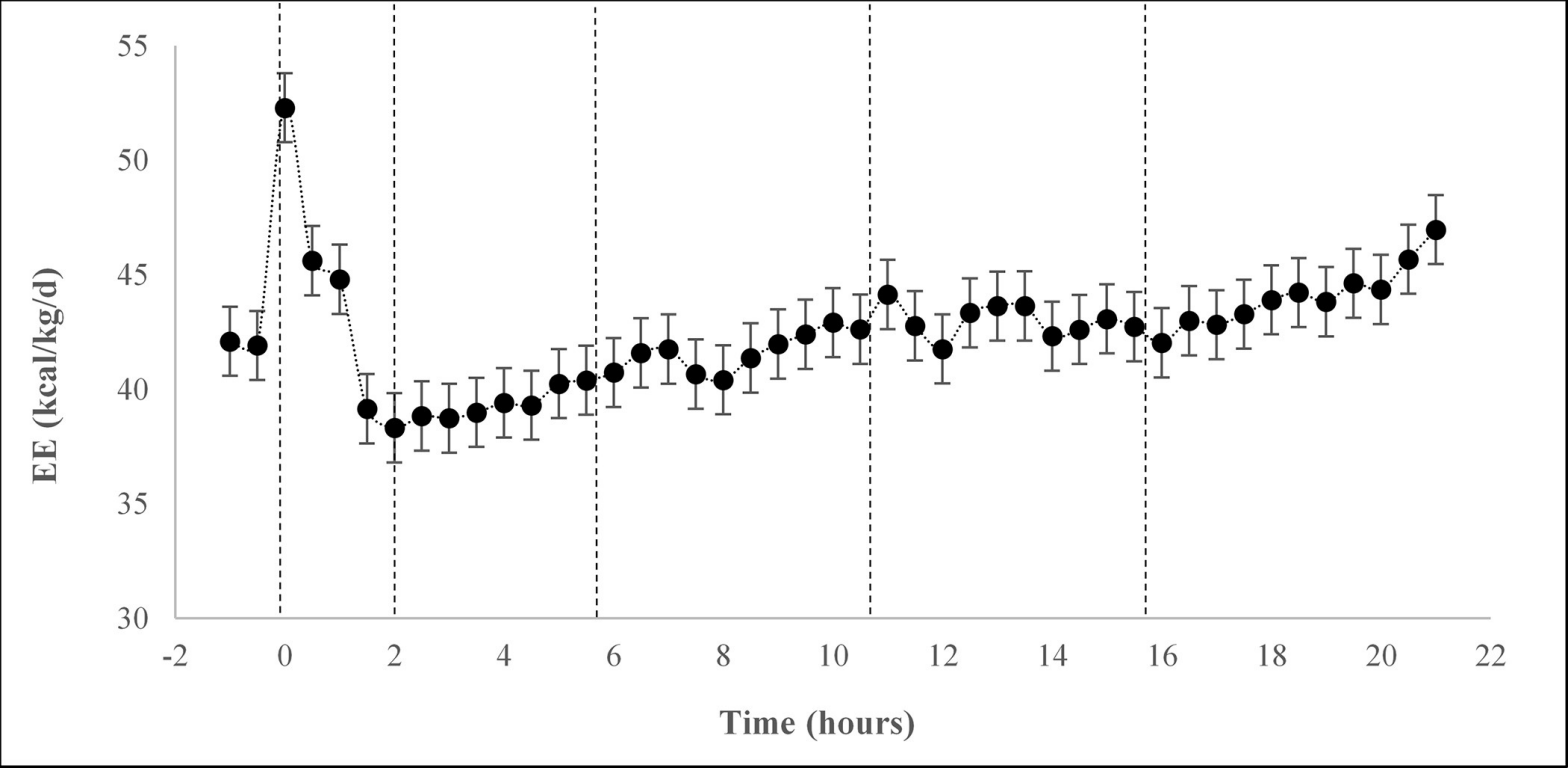
Average EE of cats (n = 19) consuming three experimental diets differing in PGR. Vertical dotted lines separate fasted, immediate postprandial, postprandial, fed, return to fasted and late fasted time points, respectively.

Following feeding, EE increased until 1.5 h, decreased between 2 and 4 h, remained constant until 20.5 hours, then increased until the end of the calorimetry period (*P* < 0.001; Fig 1). Energy expenditure did not differ between dietary treatments in the fasted state (-1–0 h; *P* = 0.160), the immediate postprandial state (0–2 h; *P* = 0.148), or the postprandial state (0–5.5 h; *P* = 0.167). There were also no differences in RFMR between dietary treatments (*P* = 0.890).

In the fed state (5.5–10.5 h), EE was highest in cats fed the HighPGR diet, intermediate in cats fed the MediumPGR diet, and lowest in cats fed the LowPGR diet (*P* < 0.001). In the return to fasted state (10.5–15.5 h), EE was higher in cats fed the HighPGR and LowPGR diets than cats fed the MediumPGR diet (Table 3; *P* < 0.001). In the late fasted state (15.5–21 h), EE was higher in cats fed the HighPGR diet compared to cats fed the Medium and LowPGR diets (*P* < 0.001). The cumulative EE over the 21 h period was higher in cats fed the HighPGR diet compared to cats fed the other two diets (*P* < 0.001).

**Table 3:** Energy expenditure in kcal/kg d^-1^ (and kJ/kg d^-1^) of cats (n = 19) consuming three commercial diets differing in PGR.

Variable	HighPGR	MedPGR	LowPGR	SEM	*P*-value
RFMR^1^	36.5 (152.7)	36.1 (151.0)	35.4 (148.1)	2.18 (9.12)	0.89
Overall (0–21 h)	43.3 (181.2)^a^	41.8 (174.9)	41.6 (174.1)	0.29 (1.21)	< 0.001
Fasted (-1–0 h)	42.1 (176.1)	40.9 (171.1)	39.8 (166.5)	1.19 (4.98)	0.160
Postprandial (0–5.5 h)	41.7 (174.5)	41.5 (173.6)	40.7 (170.3)	1.54 (6.44)	0.167
Immediate postprandial (0–2 h)	43.4 (181.6)	44.5 (186.2)	42.4 (177.4)	1.78 (7.45)	0.148
Fed (5.5–10.5 h)	42.2 (176.6)^a^	41.1 (172.0)^b^	39.9 (166.9)^c^	0.47 (1.97)	< 0.001
Return to fasted (10.5–15.5 h)	44.7 (187.0)^a^	42.4 (177.4)^b^	43.6 (182.4)^a^	0.60 (2.51)	< 0.001
Late fasted (15.5–21 h)	44.1 (184.5)^a^	41.3 (172.8)^b^	42.0 (175.7)^b^	0.56 (2.34)	< 0.001

^1^RFMR: Resting fed metabolic rate

^a–c^Within a row, means without a common superscript differ (*P* < 0.05).

For the first 2 h postprandial, the HIF per 100 g DM of diet was higher for MediumPGR than the Low- and HighPGR dietary treatments (Table 4; *P* < 0.001). For the complete calorimetry period (0–21 h), the HIF per 100 g DM of diet did not differ among dietary treatments (*P* = 0.127). Over the first 2 h postprandial, HIF amounted to 1.58%, 2.03% and 1.60% of ME intake for the High-, Medium- and LowPGR diets, respectively, and did not differ among dietary treatments (*P* = 0.130; Table 4). Over the whole calorimetry period, (0–21 h) the HIF was 21.7%, 21.6% and 19.5% of ME intake for the High, Medium and LowPGR diets, respectively, and did not differ among dietary treatments (Table 4).

**Table 4:** ME and HIF used to determine NE per 100g of diet.

Variable	HighPGR	MedPGR	LowPGR	SEM	*P*-value
ME (kcal/100g DM)^1^	458.9^a^	490.6^b^	505.5^c^	3.85	< 0.001
ME (kJ/100g DM)	1920.0^a^	2052.7^b^	2115.0^c^	16.1	< 0.001
HIF_0–2 h_^2^					
HIF (% ME)	1.58	2.03	1.60	0.25	0.130
HIF (kcal/100g DM)	5.82^a^	8.87^b^	6.56^a^	1.00	< 0.001
HIF (kJ/100g DM)	24.4^a^	37.1^b^	27.4^a^	4.18	< 0.001
Measured NE (kcal/100g DM)	453.03^a^	481.7^b^	499.0^c^	1.00	< 0.001
Measured NE (kJ/100g DM)	1895.5^a^	2015.4^b^	2087.8^c^	4.18	< 0.001
HIF_0–21 h_^3^					
HIF (% ME)	21.7	21.6	19.5	2.15	0.501
HIF (kcal/100g DM)	77.6	94.6	79.3	9.20	0.127
HIF (kJ/100g DM)	324.7	395.8	331.8	38.5	0.127
Measured NE (kcal/100g DM)	381.3^a^	396.0^b^	426.3^c^	9.20	< 0.001
Measured NE (kJ/100g DM)	1595.4^a^	1656.9^b^	1783.6^c^	38.5	< 0.001

^a–c^Within a row, means without a common superscript differ (*P* < 0.05).

^1^Analyzed in Asaro et al. [4]

^2^HIF from 0–2 h postprandial

^3^HIF from 0–21 h postprandial

### Net energy & proposed models

**Fig 2:**
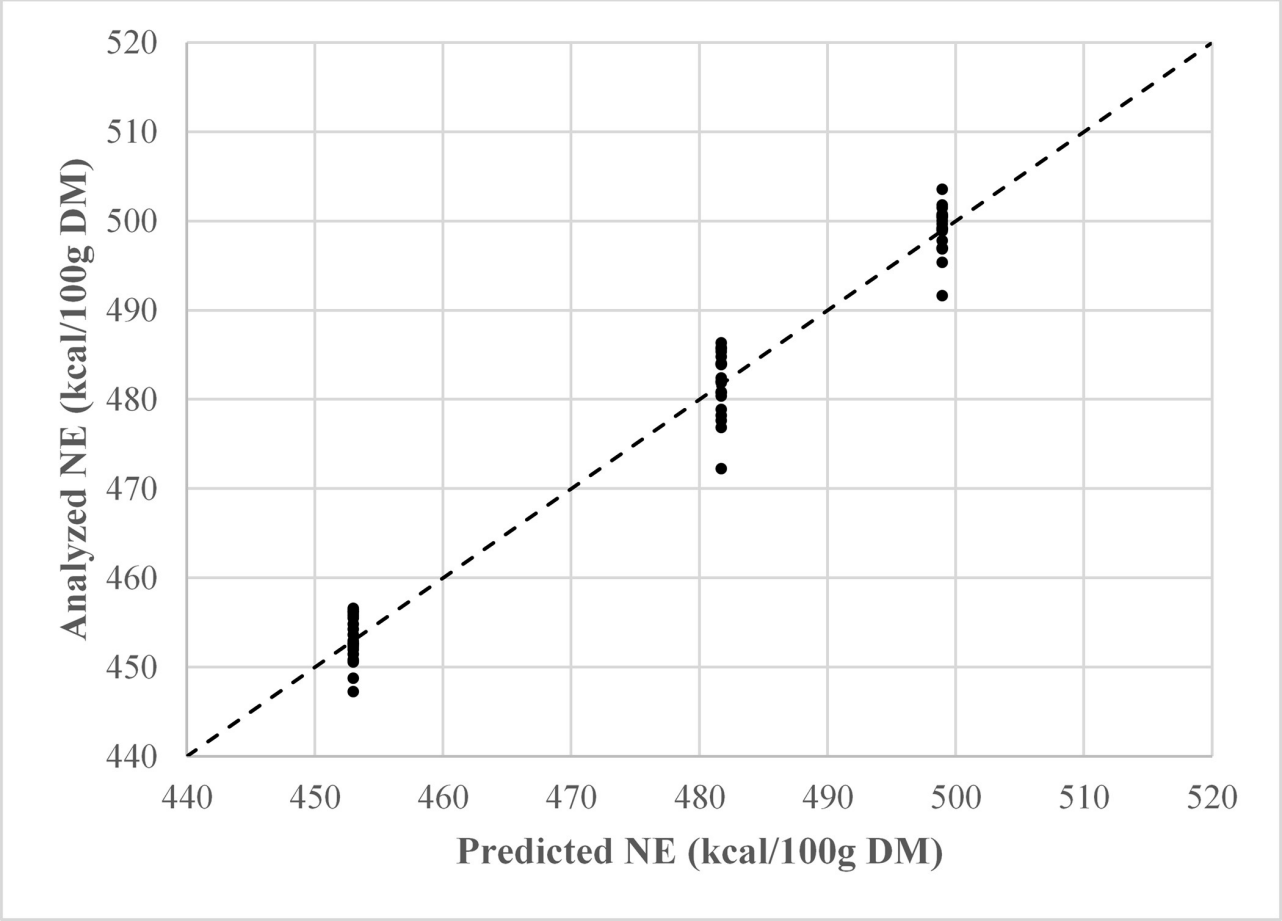
Observed versus predicted plot of dietary net energy using equation 1 (NE = (.946 × ME) + (.519 × CP)– 2.186); R^2^ = .976.

**Fig 3:**
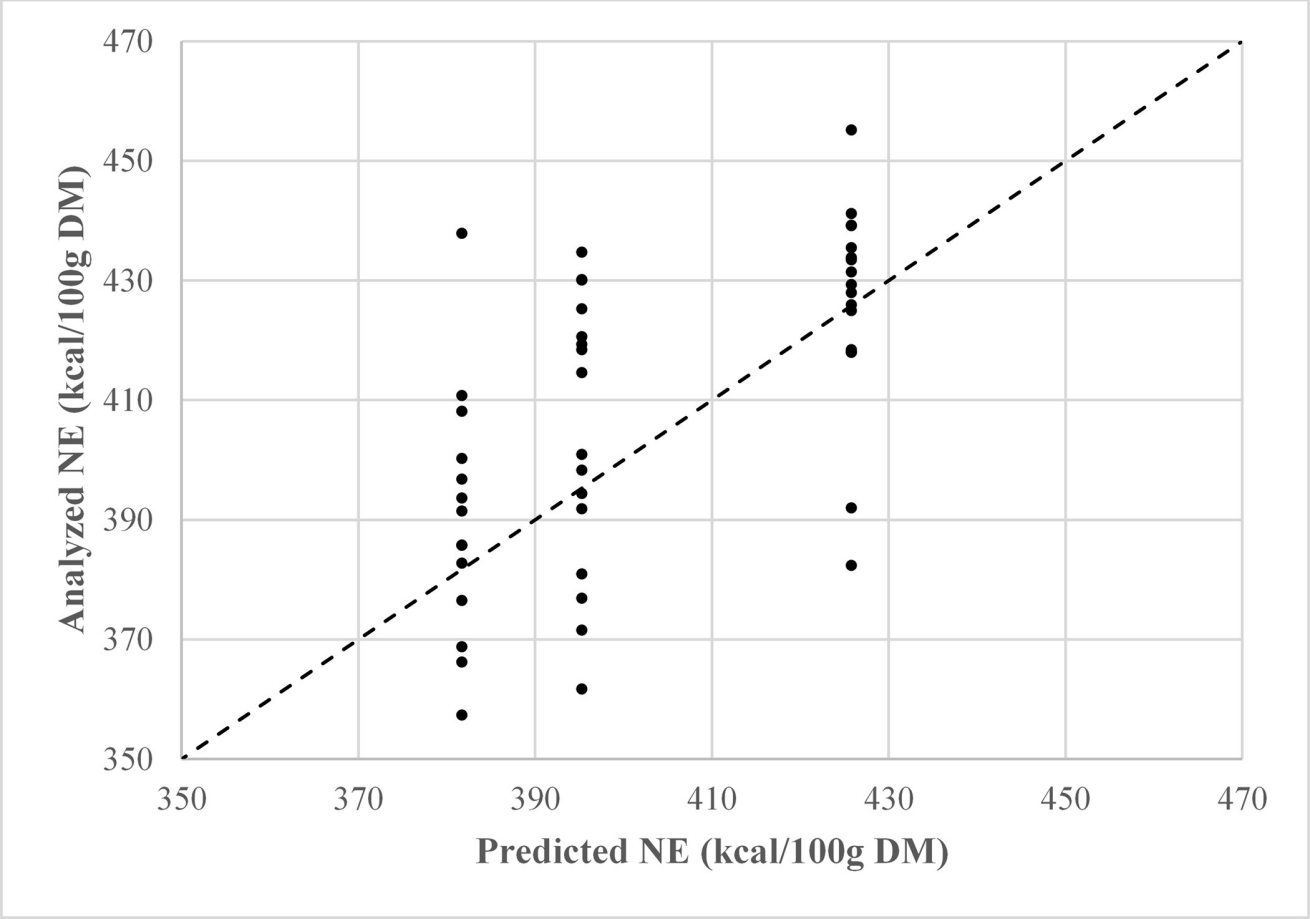
Observed versus predicted plot of dietary net energy using equation 5 (NE = (.693 × ME) + (3.375 × CP)– 74.533); R^2^ = .330.

Net energy models were created with HIF values taken from 0–2 h (Table 5; equations 1–4), and 0–21 h (Table 5; equations 5–8). There were no numerical differences in fit statistics among models which used the same set of HIF values. However, multicollinearity (VIF > 10) was detected in every model except those which included metabolizable energy and crude protein as dietary predictors (equations 1 and 5) (Table 5). Therefore, equations 1 and 5 were considered the most reliable to predict dietary net energy (Figs 2 and 3).

**Table 5:** Proposed NE models and associated R^2^ and RMSPE values using HIF values from 0–2 h and 0–21 h postprandial.

	Proposed Model	R^2^	RMSPE	ECT%^1^	ER%^2^	ED%^3^
HIF_0–2 h_	*1*. NE* *= (*.*946 × ME*^*¶*^*) + (*.*519 × CP*^*§*^*)– 2*.*186*	.976	2.99	0	2.42	97.58
	*2*. *NE = (*.*747 × ME)—(*.*752 × Starch) + 139*.*799*	.976	2.99	0	2.42	97.58
	*3*. *NE = (1*.*158 × ME)—(*.*816 × CL*^*†*^*)– 68*.*523*	.976	2.99	0	2.42	97.58
	*4*. *NE = (*.*756 × ME) + (7*.*282 × CF*^*‡*^*) + 97*.*013*	.976	2.99	0	2.42	97.58
HIF_0–21 h_	*5*. *NE = (*.*693 × ME) + (3*.*375 × CP)– 74*.*533*	.330	25.94	0.01	67.37	32.62
	*6*. *NE = —(*.*595 × ME)—(4*.*886 × Starch) + 848*.*147*	.330	25.94	0.01	67.37	32.62
	*7*. *NE = (2*.*069 × ME)—(5*.*306 × CL)– 505*.*621*	.330	25.94	0.01	67.37	32.62
	*8*. *NE = —(*.*541 × ME) + (47*.*320 × CF) + 570*.*107*	.330	25.94	0.01	67.37	32.62

^1^ECT: error in central tendency; expressed as percentage of RMSPE

^2^ ER: error due to regression; expressed as percentage of RMSPE

^3^ ED: error due to random disturbance; expressed as percentage of RMSPE

*Net energy (kcal/100g DM)

^¶^Metabolizable energy (kcal/100g DM)

^§^Crude protein (g/100g DM)

^†^Crude lipids (refers to crude fat; g/100g DM)

^‡^Crude fibre (g/100g DM)

## Discussion

The present study is the first to propose equations that estimate NE values of domestic cat diets. Though indirect calorimetry has previously been used to analyze EE, this study is original in quantifying the HIF as a proportion of ME intake in cats. Furthermore, to our knowledge, this is the first study to follow cats for a prolonged period of time post-feeding and determine changes in energy expenditure at different points throughout the 21 h calorimetry period. The results of this study were novel and resulted in the development of equations that have potential to drastically alter how we determine dietary energy and the resultant feeding recommendations for adult cats.

Basal metabolic rate (**BMR**) could not be measured in the current experiment. Measurements of BMR require an animal to be in a postabsorptive state, and not have undergone significant activity [17]. However, eliminating activity in an animal study requires restraining of animals, which would thereby increase stress and lead to inaccurate measurements. Thus, RFMR was used to approximate BMR, which has been defined as the lowest observed value of energy expended by an animal in a fed state that otherwise meets the criteria for basal metabolism [3].

The observed measurements of EE were expected and are similar to those reported in literature [18–22]. In 16 neutered normal-weight cats, the least observed metabolic rate (**LOMR**) was approximately 39 ± 1 kcal/kg d^-1^ [19]. The LOMR can be described as the lowest metabolic rate observed during a continuous record of metabolic rate and is synonymous to our RFMR measurements [19, 23]. In cats fed both high fat and high carbohydrate diets, fasted EE ranged from 44–47 kcal/kg d^-1^, and fed EE ranged from 43–51 kcal/kg d^-1^ [18]. Furthermore, Center et al. [20] found daily resting EE to be 36 ± 7.7 kcal/kg d^-1^ in cats with underweight, normal and overweight body condition scores. These similarities provide support for our results and validate their use to predict energy algorithms for the domestic cat in general.

The duration of observed postprandial EE increase in the present study is similar to that of other species, both monogastric and ruminant. In sheep, EE increased by 40–80% during consumption of a meal and persisted for up to 2 h before rapidly declining to rates similar to those recorded in a fasted state [24]. In fur seals and sea lions, metabolism peaked approximately 3 h after a meal, and returned to fasting levels between 6 and 10 h [25]. Furthermore, dietary induced thermogenesis in humans lasted for 4.8 h and 5.8 h after consumption of processed-food meals and whole-food meals respectively [26]. Certainly, variances in animal size, meal composition, and experimental methods may contribute to differences in HIF among species. Thus, it was not surprising to see a 2 h postprandial energy response in cats in the present study.

Heat increment of feeding values from 0–2 h were lower than expected compared to terrestrial herbivores and omnivores. Although HIF values have not been previously reported for the domestic cat, the HIF accounts for up to 30% of ME intake in other mammals and birds [27]. However, in other carnivorous endotherms, HIF values appear to be much lower; in house wrens [28], harbor seals [29], and penguin chicks [30], the HIF amounted to 6.3%, 4.7%, and 10.0% of ingested ME, respectively. Furthermore, Kendall et al. [31] found that cats have significantly lower costs to maintain body weight versus beagles when compared on a metabolic body weight basis [11]. Interestingly, HIF values in this study were similar to those reported in carnivorous fish; in salmonids fed complete diets, the HIF was less than 3% of ingested ME [27]. Furthermore, due to the low energy cost of protein metabolism in fish, the NE of protein is higher for salmonids than mammals and birds [27]. This similarity suggests that perhaps a high efficiency of utilization, and thus low energy production in the postprandial state, results in analogous HIF values among carnivorous species. Perhaps like carnivorous fish, cats have a low energy cost of protein metabolism [27]. The unique relationship between protein metabolism and energy production in cats may explain why our HIF values are considerably lower than those reported in other mammals, but similar to carnivorous fish.

When HIF was taken from 0–21 h and expressed as a proportion of ME, the present results were comparable to those in other mammals. In pigs, the HIF for starch, sugars, and digestible crude protein represented 29% of ME content on average [1]. In ruminants, the HIF is variable and can represent 30–70% of ME intake, though microbial fermentation and rumination contribute largely to these costs [32]. However, by taking the complete calorimetry period into account, measurements cannot necessarily be categorized as HIF, which are usually measured in a postprandial rather than a fasted state. Furthermore, models (equations 5–8) created using the HIF values from 0–21 h fit our data poorly as shown by the low R^2^ values. Due to the amount of data collected and the variation in the data over the 21 h postprandial period, it was difficult to achieve a good fit while being limited to the inclusion of 2 dietary parameters. Evaluating a greater number of experimental diets would allow for the fitting of more parameters, which could potentially result in equations that more accurately fit the observed NE values. Regardless, it is suggested that the observed increase in EE at the end of the calorimetry period is a characterizing feature of cat nutrient metabolism in general, as it was consistently observed among all diets. As previously mentioned, the costs associated with protein metabolism are higher compared to other macronutrients [33]. It is possible that the cats used in this study displayed increased rates of protein oxidation or deposition at the end of the calorimetry period. Investigation of the timing and specifics of nutrient metabolism in cats may elucidate this unexplained increase in EE.

This study was limited in the ability to parameterize variables in our models. Net energy prediction equations have been developed for other species that parameterize all the dietary macronutrients in a single equation [1, 2]. However, as the present study only employed the use of three diets, only one macronutrient input, in combination with ME, could be parameterized at a time. Because of these limitations, fit statistics did not differ among models using the same set of HIF values. However, assessment for multicollinearity determined that predictor variables were highly correlated in every model except those which included metabolizable energy and crude protein as dietary parameters (equations 1 and 5). It is likely that this issue is reflective of issues in the sampling methodology as data has been collected from a small subset of independent variables, and not multicollinearity within the population. Regardless, multicollinearity can create inaccurate estimates of regression coefficients and degrade the predictive ability of a model [14]. Therefore, we cannot be confident that the parameterized coefficients or fit statistics of the multicollinear models are accurate. Indeed, the authors of the present study investigated methods such as ridge regression and lasso regression to combat issues of multicollinearity. However, due to the likelihood that the issue is due to sampling methodology, these methods were not implemented. Obtaining an expanded data set with multiple experimental diets would likely rectify the problem. As such, the equations which included metabolizable energy in combination with crude protein (equations 1 and 5) were determined to be the most reliable models to predict dietary net energy. Additionally, this study was the first to express HIF as a proportion of ME for cats, therefore restricting the ability to comment on the accuracy the developed prediction equations until more work is completed. Thus, further research would focus on testing the accuracy of these equations to predict NE values of additional commercial diets.

## Conclusion

In conclusion, NE, which accounts for energy spent in the digestion, absorption and metabolism of nutrients, is a more accurate measure of energy directly available to an animal [3]. Expressing energy density on an NE basis could allow a more accurate feeding recommendation than what is currently utilized for commercial feeding recommendations and limit the provision of excess calories to cats. Thus, the present study proposed multiple equations to estimate a NE value and recommends using the dietary parameters of ME and CP to predict NE of complete diets for domestic cats.

## Supporting information

S1 Table Analyzed nutrient composition of the 3 experimental diets differing in perceived glycemic response (PGR). (PDF)

S2 Table Body weight and intake of energy for cats consuming 3 experimental diets. (PDF)
