# Supplementary material for: Modelling net energy of commercial cat diets
Source: PLoS One. 2019 Jun 11;14(6):e0218173. doi: 10.1371/journal.pone.0218173 (PMC6559639; doi:10.1371/journal.pone.0218173)
Supplement: S1 Table — (PDF) [file pone.0218173.s001.pdf]

Table 1. Analyzed nutrient composition of the 3 experimental diets differing in perceived glycemic response (PGR)[1].

Item	HighPGR[2]	MediumPGR[3]	LowPGR[4]
Moisture, %	7.16	6.76	5.31
Ash, %	6.36	6.31	6.38
Crude protein[5], %	38.02	35.86	42.06
Crude fat, %	10.83	20.02	20.42
Nitrogen-free extract[6], %	34.1	29.5	23.6
Starch[7], %	36.75	30.72	23.56
Crude fiber, %	1.17	1.78	2.58
Acid detergent fiber, %	1.88	2.95	2.43
Neutral detergent fiber, %	7.36	12.58	10.57
Available lysine, %	1.62	1.91	2.80
GE, kcal/kg	4916	5253	5462
Calculated ME[8]	3752	4081	4137

[1] Each diet was analyzed in triplicate. Results (except moisture) presented on a dry-matter basis. [2] HighPGR was Purina ONE Chicken and Rice (Nestlé, St. Louis, MO, USA) containing as main ingredients: chicken, brewer's rice, corn gluten meal, poultry by-product meal, wheat flour, animal fat preserved with mixed-tocopherols, whole grain corn, soy protein isolate, fish meal, animal liver flavor, KCl, H_3PO_4, $CaCO_3$, caramel color, choline chloride, and salt. [3] MediumPGR was Iams Kitten Proactive Health (Procter & Gamble, Cincinatti, OH) containing as main ingredients: chicken, chicken by-product meal, corn meal, chicken fat preserved with mixed tocopherols, dried beet pulp, ground whole grain sorghum, dried egg product, natural flavor, fish oil preserved with mixed tocopherols, KCl, fructooligosaccharides, choline chloride, $CaCO_3$, brewer's dried yeast, DL-Met, and salt. [4] LowPGR was Innova (Procter & Gamble, Cincinatti, OH) containing as main ingredients: turkey, chicken, chicken meal, whole grain barley and whole grain brown rice, chicken fat preserved with mixed tocopherols, peas, natural flavors, apples, herring, flaxseed, eggs, blueberries, pumpkin, tomatoes, sunflower oil, KCl, DL-Met, carrots, pears, cranberries, menhaden oil, cottage cheese, taurine, green beans, alfalfa sprouts, parsnips, and salt. [5] Percentage N $\times$ 6.25. [6] NFE (%) = 100 − moisture (%) − protein (%) − fat (%) − fiber (%) − ash (%). [7] Determined using AOAC Official Method 979.10. [8] Calculated with modified Atwater equation (AAFCO, 1997): ME (kcal/kg) = 10 $\times$ (3.5 $\times$ Crude Protein % + 8.5 $\times$ Crude Fat % + 3.5 $\times$ Nitrogen-Free Extract %).
