# Supplementary material for: Modelling net energy of commercial cat diets
Source: PLoS One. 2019 Jun 11;14(6):e0218173. doi: 10.1371/journal.pone.0218173 (PMC6559639; doi:10.1371/journal.pone.0218173)
Supplement: S2 Table — (PDF) [file pone.0218173.s002.pdf]

Table 2. Body weight and intake of energy for cats consuming 3 experimental diets

Variable	High PGR	Med PGR	Low PGR	SEM[1]	*P*-value
BW, (kg)	4.99	4.95	4.94	0.34	0.99
Intake, (g/d)	45.6[a]	37.9[b]	40.0[b]	2.68	0.02
Calculated ME[2] Intake, (kcal/d)	155.8	154.5	154.3	10.08	0.98
True ME Intake[3], (kcal/d)	230.7[a]	173.9[b]	196.2[b]	12.71	<0.001

[a-c]Within row, means without a common superscript differ ($P < 0.05$).

[1]Means were based on 19 cat observations per diet.

[2] Calculated with modified Atwater equation (AAFCO, 1997): ME (kcal/kg) = [3.5 × CP (%) + 3.5 × NFE (%) + 8.5 × crude fat (%)] × 10.

[3] Using analyzed ME from Asaro et al., 2017.
